# Hypocomplementemia is associated with more severe renal disease and worse renal outcomes in patients with ANCA-associated vasculitis: a retrospective cohort study

**DOI:** 10.1080/0886022X.2020.1803086

**Published:** 2020-08-13

**Authors:** Aglaia Chalkia, Konstantinos Thomas, Panagiota Giannou, Alexandros Panagiotopoulos, Emilia Hadziyannis, Athanasia Kapota, Harikleia Gakiopoulou, Dimitrios Vassilopoulos, Dimitrios Petras

**Affiliations:** a Nephrology Department, Hippokration General Hospital, Athens, Greece; b 2nd Department of Medicine and Laboratory, Clinical Immunology – Rheumatology Unit, School of Medicine, National and Kapodistrian University of Athens, Athens, Greece; c 1st Department of Pathology, School of Medicine, National and Kapodistrian University of Athens, Athens, Greece

**Keywords:** ANCA, complement, glomerulonephritis, vasculitis, end-stage renal disease

## Abstract

**Background:**

The complement system has been recently proposed to play an important role in the pathogenesis of ANCA-associated vasculitis (AAV). This study evaluated the value of serum and kidney deposited C3 in predicting renal outcomes in AAV.

**Methods:**

This was a retrospective study of 47 patients with AAV, who were categorized according to their serum C3 levels as hypo- or normo-complementemic and to those with positive or negative kidney biopsy immunofluorescence (IF) for C3. Baseline characteristics as well as progression to end-stage renal disease (ESRD) between the 2 groups were compared.

**Results:**

In total, 23% (11/47) were hypo-complementemic; these patients were older (74 vs. 65 years, *p* = 0.013), had higher creatinine levels (4.9 vs. 2.2 mg/dL, *p* = 0.006), were more often hemodialysis dependent (64% vs. 19%, *p* = 0.009) and progressed more often to ESRD (55% vs. 11%, *p* = 0.01) compared to normo-complementemic patients (*n* = 36). On multivariate analysis, serum creatinine at diagnosis (HR = 16.8, 95%CI: 1.354–208.62, *p* = 0.028) and low serum C3 (HR = 2.492; 95% CI: 1.537–11.567; *p* = 0.044) were independent predictors for ESRD. Among 25 patients with an available kidney biopsy, 56% had C3 deposition by IF and displayed more often a mixed histological pattern (72% vs. 27%, *p* = 0.033), low serum C3 levels (42% vs. 18%, *p* < 0.001) and serious infections during follow-up (57% vs. 18%, *p* = 0.047) compared to those with negative (*n* = 11) IF staining.

**Conclusion:**

Almost one of four patients with AAV has low C3 levels at diagnosis which is associated with more severe renal disease and worse renal outcomes (ESRD). This should be taken into account in therapeutic and monitoring strategies.

## Introduction

Anti-neutrophil cytoplasmic autoantibody (ANCA)-associated vasculitis (AAV) is a systemic small vessel vasculitis with multiorgan involvement and significant associated morbidity and mortality. The role of complement activation, mainly of the alternative pathway, in its pathogenesis has been recently suggested to be significant [1]. Although necrotizing and crescentic glomerulonephritis related to ANCAs is typically referred as a “pauci-immune” process, in certain cases there is immune complex and complement deposition in glomeruli as detected by immunofluorescence (IF) [2]. Kidney complements deposits of patients with AAV correlated with greater kidney damage, more significant proteinuria, and overall disease activity [3]. Nevertheless, data regarding the prognostic significance of low C3 levels and C3 kidney deposition in patients with AAV are limited.

Hence, the aim of this study was to investigate the potential correlation of C3 in the serum and kidneys with renal characteristics and prognosis in a cohort of patients with AAV.

### Patients and methods

This was a retrospective cohort study of patients with AAV followed in a tertiary referral hospital (Nephrology Department and Clinical Immunology––Rheumatology Unit, 2nd Department of Medicine, Hippokration General Hospital, Athens, Greece) through the years 2002 and 2019. Among 62 patients of the Caucasian race with newly diagnosed AAV, 47 patients with available complement levels at diagnosis were included in the study. All patients with AAV satisfied the inclusion criteria of ACR 1990 or the Chapel Hill Consensus Conference definitions [4] for granulomatosis with polyangiitis (GPA), microscopic polyangiitis (MPA), and ANCA-limited renal vasculitis (LRV). Patients with other underlying diseases that cause secondary vasculitis, such as systemic lupus erythematosus, rheumatoid arthritis, IgA nephropathy, Sjogren’s syndrome, malignancy and Behcet’s disease were excluded clinically and serologically. There was no temporal relationship between clinically evident vasculitis and the administration of an offending drug. So, drug-induced vasculitis was also excluded. The patients, who included in the study, met one of the two following criteria: 1. Necrotizing vasculitis predominantly affecting small vessels and/or necrotizing glomerulonephritis with few or no immune deposits 2. positive ANCA with IF and ELISA. The study was approved by the Hippokration General Hospital’s institutional review board (57/26-3-2018) and the consent of all patients for their retrospective chart review was obtained.

Demographic data were collected for each patient (age, sex, type of AAV, and patient follow-up). Clinical characteristics at diagnosis, such as major organ involvement (lung: pulmonary hemorrhage, kidney: hemodialysis dependence) and induction treatment (with or without plasmapheresis), episodes of relapses, and serious infections during follow-up and eventually mortality.

As far as laboratory results, hemoglobin, creatinine level, 24-h proteinuria, hematuria, ANCA status (p-ANCA MPO, c-ANCA PR3, double positivity [anti-GBM with ANCA] or negative), serum C3, C4 and ANA, and anti-dsDNA were included. C3 and C4 levels were assessed by tholosimetry. Anti-neutrophil cytoplasmic antibodies (c-ANCA and p-ANCA) were determined by a direct IF technique and the specificity for PR3 and MPO by ELISA. The serum C3 reference range in our laboratory is 75–180 mg/dL. The patients were divided into 2 groups, those with low C3 (hypo-complementemic: <75 mg/dL), and those with normal C3 (normo-complementemic: 75–180 mg/dL). No one had C3 levels higher than 180 mg/dL.

A total of 40 patients presented with renal involvement, which was defined by the presence of 24-h urine proteinuria of >1 g, active urinary sediment (leukocyturia > 5 white blood cells/field, hematuria >5 erythrocytes/field, and/or cellular casts) and/or increase of creatinine values over 1.2 mg/dL. Kidney biopsy was performed in 25 patients (62.5%). Renal histology was evaluated using light microscopy (hematoxylin and eosin, periodic acid-Schiff, and Masson trichrome stains) and was classified into 4 histological subtypes according to the 2010 histologic classification of AAV [5]. Those presenting ≥50% of all glomeruli globally sclerosed were screlotic type and those with ≥50% normal glomeruli were classified as focal type. Those showing ≥50% of glomeruli with semilunar cells were classified as crescentic type and those not presenting the anterior criteria were classified as mixed type. The presence of immunoglobulin (IgG, IgM, IgA), complement (C3, C1q, C4), kappa and lambda light chains was evaluated by direct IF on the fresh frozen tissue. The staining of C3 was routinely performed using polyclonal rabbit anti-human antiserum, at dilution 1:50 (Dako, Copenhagen, Denmark). According to their C3 deposition for at least 1+ in a 0–4+ scale, patients were divided into those with (+) and (–) IF. Electron microscopy was not performed.

### Statistical analysis

Data were analyzed using Statistical Package for the Social Sciences (SPSS) software program, version 19.0 for Windows (SPSS, Chicago, IL, USA). Values are expressed as medians and interquartile ranges (IQR) for continuous variables and percentages for categorical variables. Comparison of variables was done using the independent samples t-Test for Continuous variables and Chi-square test or Fisher’s exact test for categorical variables. A two-sided value *p* < 0.05 was considered as statistically significant. To address the independent predictive value of factors associated with the progression to end-stage renal disease (ESRD––defined as eGFR < 15 mL/min/1.73 m^2^ or requiring hemodialysis) a univariate and multivariate Cox proportional hazards regression analysis were performed. We used the variables with p values of less than 0.1 between progressors to ESRD and non-progressors.

## Results

### Baseline patient characteristics

Forty-seven patients with AAV were included; 53% (25/47) were men, with a median age of 67 years (Table 1). The most frequent type of vasculitis was GPA (55%), followed by MPA (36%) and LRV (9%). The majority of patients were ANCA+ (94%), most of them with MPO specificity (57%).

**Table 1.** Baseline patient characteristics, therapy, and disease outcomes according to their serum C3 levels.

	Total	Hypo-complementemic (C3*s* < 75 mg/dL)	Normo-complementemic (C3 > 75 mg/dL)	*p-*value
	*n* = 47	*n* = 11	*n* = 36	
Age, years, median (IQR)	67 ( 55.7–75.9)	74 (66.3–80.4)	64.6 (53.5–73.2)	0.013
Males, *n* (%)	25 (53%)	6 (55%)	19 (54%)	0.918
AAV type				0.291
GPA, *n* (%)	26 (55%)	5 (45%)	22 (61%)	
MPA, *n* (%)	17 (36%)	6 (55%)	10 (28%)	
RLV, *n* (%)	4 (9%)	0 (0%)	4 (11%)	
ANCA status				0.089
Anti-MPO+, *n* (%)	27 (57.4%)	5 (45%)	22 (61%)	
Anti-PR3+, *n* (%)	15 (32%)	3 (27%)	12 (33%)	
Double +, *n* (%)	2 (4.2%)	2 (18%)	0 (0%)	
Negative, *n* (%)	3 (6.4%)	1 (9%)	2 (6%)	
Low serum C4 mg/dL, *n* (%)	1 (2%)	1 (9%)	0 (0%)	0.234
ANA, anti-DNA positive, *n* (%)	0 (0%)	0 (0%)	0 (0%)	1
Organ involvement				
Cutaneous signs, *n* (%)	7 (15%)	1 (9%)	6 (17%)	1
Ear, nose, throat, *n* (%)	14 (30%)	3 (27%)	11 (31%)	0.835
Peripheral Nervous System, *n* (%)	12 (26%)	1 (9%)	11 (31%)	0.413
Joints, *n* (%)	19 (40%)	3 (27%)	16 (44%)	0.732
Lung involvement, *n* (%)	32 (68%)	10 (91%)	22 (61%)	0.078
Pulmonary Hemorrhage, *n* (%)	6 (13%)	3 (27%)	3 (8%)	0.131
Kidney involvement, *n* (%)	40 (85%)	11 (100%)	29 (81%)	0.175
Hematuria, *n* (%)	24 (51%)	7 (64%)	17 (47%)	0.636
Proteinuria, > 1g/24h *(data from 32 patients)*	18/32 (56%)	8//10 (80%)	10/22 (45%)	0.26
**Serum creatinine** **(mg/dL), media** *n* (**IQR)**	**2 (1–4.4)**	**5.2 (3–6.5)**	**1.25 (0.9–3)**	**0.006**
**Hemodialysis,** *n* (**%)**	**14 (30%)**	**7 (64%)**	**7 (19%)**	**0.009**
Kidney biopsy (among 40 patients with renal involvement)	25/40 (62.5%)	8/11 (73%)	17/29 (59%)	0.486
Histological type				0.173
Mixed, *n* (%)	13 (52%)	4 (50%)	9 (53%)	
Focal, *n* (%)	7 (28%)	1 (13%)	6 (35%)	
Sclerotic, *n* (%)	3 (12%)	1 (13%)	2 (12%)	
Crescentic, *n* (%)	2 (8%)	2 (25%)	0 (0%)	
Positive IF for C3, *n* (%)	14/25 (56%)	6/8 (75%)	8/17 (47%)	0.234
Induction Therapy (*n* = 47)				0.624
CYC, *n* (%)	22 (47%)	7 (64%)	15 (42%)	
RTX, *n* (%)	15 (32%)	2 (18%)	13 (36%)	
CYC + RTX, *n* (%)	6 (13%)	2 (18%)	4 (11%)	
MTX/MMF, *n* (%)	4 (8%)	0 (0%)	4 (11%)	
Plasmapheresis, *n* (%)	9 (19%)	4 (36%)	5 (14%)	0.183
Maintenance Therapy (*n* = 36)*				0.218
RTX, *n* (%)	29 (62%)	8 (73%)	21 (58%)	
AZA, *n* (%)	4 (9%)	1 (9%)	3 (8%)	
MMF, *n* (%)	3 (6%)	1 (9%)	2 (5%)	
Relapses, *n* (%)	18 (38%)	3 (27%)	15 (42%)	0.299
**ESRD, *n* (%)**	**10 (21%)**	**6 (55%)**	**4 (11%)**	**0.01**
Mortality, *n* (%)	8 (17%)	2 (18%)	6 (17%)	1
**ESRD and/or mortality, *n* (%)**	**12 (26%)**	**6 (55%)**	**6 (17%)**	**0.02**

Values are expressed as numbers of patients (*n*), or median interquartile ranges (IQR). AAV, ANCA associated vasculitis; GPA, granulomatosis with polyangiitis; MPA, microscopic polyangiitis; RLV, renal-limited vasculitis; ANCA antineutrophil cytoplasmic antibodies; MPO, myeloperoxidase; PR3, proteinase 3; Double positive, anti-GBM (+) and ANCA (+); C4, complement 4; IF, immunofluorescence; CYC, cyclophosphamide; RTX, rituximab; MTX, methotrexate; MMF, mycophenolate mofetil; AZA, azathioprine; ESRD, end-stage renal disease.

Statistical significant differences are shown in bold (*p* < 0.05).

*Four patients who died in the induction phase and seven patients without detailed data available were excluded.

Forty patients (85%) had renal involvement at diagnosis and approximately one third (30%, 14/47) required hemodialysis. The median serum creatinine at diagnosis was 2 mg/dL while 56% (18/32) of patients had significant proteinuria (>1 g/24h) (32 patients with available data concerning proteinuria). In 25 patients a kidney biopsy was performed. Approximately half (52%) of patients had a mixed histologic pattern, followed by focal (28%), sclerotic (12%) and crescentic (8%) types.

In 68% of the patients, who included, presented with lung involvement and among them 6 (13%) presented with pulmonary hemorrhage (Table 1).

### Treatment and disease outcomes

The induction therapeutic regimen consisted of glucocorticoids (three daily pulses of methylprednisolone 1000 mg followed by prednisolone 1 mg/kg per day with gradual dose taper) and immunosuppressives (cyclophosphamide––CYC: 47%, rituximab––RTX: 32%, CYC + RTX: 13%, mycophenolate mofetil––MMF or methotrexate––MTX: 8%, Table 1). In 19% plasmapheresis was added to the induction regimen.

Patients were followed for a median time of 56 months (range: 4-204 months). Among patients who received maintenance therapy (*n* = 36, Table 1), the majority received RTX (62%) followed by AZA (9%) or MMF (6%). During the follow-up period, 38% of patients (18/47) developed relapses (minor and major). Among them, nine patients received RTX, two patients CYC, and two patients CYC + RTX as rescue therapy.

Finally, 10 patients (21%) progressed to ESRD and 8 (17%) died (4 during the induction and 4 during the follow-up period). The causes of death were serious infections in 3 patients, lung cancer in 1 patient, heart disease in 1 and unknown causes in 4 patients. It is noteworthy that the two patients with double positivity (ANCA and anti-GBM) at diagnosis progressed to ESRD between 3-6 months of disease duration.

### Differences between hypo- and normo-complementemic patients

At baseline, 23% (11/47) of the patients had low C3 levels (<75 mg/dL, Table S1 in Supplementary Material). Compared to normo-complementemic patients, those with low C3 were older (median age 74 vs. 64.6 years, *p* = 0.013), had higher serum creatinine at diagnosis (5.2 vs. 1.25 mg/dL, *p* = 0.006), required more often hemodialysis at diagnosis (64 vs. 19%, *p* = 0.009), and progressed more frequently to ESRD (55 vs. 11%, *p* = 0.01) (Table 1).

### Differences between C3 kidney positive and negative patients

Kidney biopsies were available in 25/40 (62.5%) patients with renal involvement. Compared to patients without kidney biopsy (*n* = 15), those with renal biopsy available had more frequently significant proteinuria and higher serum creatinine at diagnosis (Table 2).

**Table 2.** Comparison of patients with renal involvement with or without kidney biopsy available.

	Patients with renal biopsy	Patients without renal biopsy	*p-*value
	*n* = 25	*n* = 15	
Age, years, median (IQR)	70.8 (64.2–77.8)	67.9 (56.4–75.4)	0.345
Males, *n* (%)	16 (64%)	6 (40%)	0.550
AAV type			0.551
GPA, *n* (%)	14 (56%)	7 (47%)	
MPA, *n* (%)	9 (36%)	6 (40%)	
RLV, *n* (%)	2 (8%)	2 (13%)	
ANCA status			0.081
Anti-MPO+, *n* (%)	17(68%)	8 (53%)	
Anti-PR3+, *n* (%)	4 (16%)	6 (40%)	
Double +, *n* (%)	2 (8%)	0 (0%)	
Negative, *n* (%)	2 (8%)	1 (7%)	
Lung involvement, *n* (%)	21 (84%)	8 (53%)	0.056
Pulmonary hemorrhage, *n* (%)	5 (20%)	1 (6%)	0.428
Kidney involvement			
Hematuria, *n* (%)	19 (76%)	5 (33%)	1
**Proteinuria > 1 g/24h**	**17/21 (81%)**	**2/11 (18%)**	**0.044**
**(data from 32 patients)**			
**Serum creatinine**	**4 (2–5.5)**	**1.4 (0.9–2.4)**	**0.020**
**(mg/dL), median (IQR)**			
Hemodialysis at diagnosis, *n* (%)	10 (40%)	4 (27%)	0.199
Low serum C3, *n* (%)	8 (32%)	3 (20%)	0.473
Plasmapheresis, *n* (%)	7 (28%)	2 (13%)	0.151
Relapse, *n* (%)	9 (36%)	6 (40%)	0.478
ESRD, *n* (%)	8 (32%)	2 (13%)	0.113
Infections, *n* (%)	10 (40%)	5 (33%)	0.534
Mortality, *n* (%)	5 (20%)	3 (20%)	0.705

Values are expressed as numbers of patients, or median interquartile ranges (IQR). AAV, ANCA associated vasculitis; GPA, granulomatosis with polyangiitis; MPA, microscopic polyangiitis; RLV, renal-limited vasculitis; ANCA antineutrophil cytoplasmic antibodies; MPO, myeloperoxidase; PR3, proteinase 3; Double positive, anti-GBM (+) and ANCA (+); C3, complement 3 level; ESRD, end-stage renal disease. Statistical significant differences are shown in bold (*p* < 0.05).

Among those 25 kidney biopsies, more than half (56%, 14/25) had C3 deposits by IF. C3 staining, mainly in mesangium, was relatively mild (≤2) in all patients. Furthermore, 43% (6/14) of C3 (+) biopsies also showed mild IgM staining (1+). The IF of the other immunoglobulins and complement components were negative. Additionally, thrombotic microangiopathy was not detected as a pattern of injury in kidney biopsies. Patients with C3 deposition (*n* = 14) as a group, had more often low C3 levels (43 vs. 18%, *p* = 0.001), a mixed histologic pattern in kidney biopsy (71 vs. 27%, *p* = 0.033) and a higher rate of serious infections during follow-up (57 vs. 18%, *p* = 0.047) compared to those without C3 deposition (*n* = 11) (Table 3).

**Table 3.** Clinical and histological characteristics of patients according to their kidney C3 deposition by IF.

	C3 IF (+/++)	C3IF negative	*p-*value
	(*n* = 14)	(*n* = 11)	
Age, years, median (IQR)	71.1 (64.5–79)	70.8 (61–73.6)	0.975
Creatinine mg/dL, median (IQR)	3.35 (2.1–5.8)	4 (1.2–5.2)	0.557
**Low serum C3, *n* (%)**	**6 (43%)**	**2 (18%)**	**0.001**
Histological type			**0.033**
**Mixed, *n* (%)**	**10 (72%)**	**3 (27%)**	
Crescentic, *n* (%)	1 (7%)	1 (9%)	
Focal, *n* (%)	1 (7%)	6 (55%)	
Sclerotic, *n* (%)	2 (14%)	1 (9%)	
IgM IF (+)	6 (43%)	3 (27%)	0.065
Hemodialysis, *n* (%)	5 (36%)	5 (45%)	0.697
Plasmapheresis, *n* (%)	5 (36%)	2 (18%)	0.407
Lung involvement, *n* (%)	12 (86%)	9 (82%)	1
Pulmonary hemorrhage, *n* (%)	3 (21%)	2 (18%)	1
**Serious infections,** *n* (**%)**	**8 (57%)**	**2 (18%)**	**0.047**
Relapses, *n* (%)	5 (36%)	4 (36%)	0.94
ESRD, *n* (%)	5 (36%)	3 (27%)	0.69
Mortality, *n* (%)	3 (21%)	2 (18%)	1
ESRD and /or mortality, *n* (%)	6 (43%)	4 (36%)	0.89

Values are expressed as numbers of patients, or median interquartile ranges (IQR). C3, complement 3 level; IgM IF, immunoglobulin M immunofluorescence; ESRD, end-stage renal disease; IF, immunofluorescence. Statistical significant differences are shown in bold (*p* < 0.05).

### Factors associated with progression to ESRD

Ten patients (21%) progressed to ESRD during follow-up. Compared to patients who did not progress to ESRD (*n* = 37), these patients (*n* = 10) were older (73 vs. 58 years, *p* = 0.04), had higher creatinine level (5.9 vs 1.6 mg/dL, *p* = 0.000), proteinuria > 1 g/24 h (70% vs. 30%, *p* = 0.120) and lower serum C3 levels (60% vs 14%, *p* = 0.006), had more often double positivity [anti-GBM with ANCA] (20% vs. 0%, *p* = 0.014) at diagnosis and sclerotic histological pattern (30% vs 0%, *p* = 0.03), more frequently required hemodialysis at diagnosis (70% vs 8%, *p* = 0.000) and developed serious infection at follow-up (50% vs 32%, *p* = 0.045).

Univariate Cox regression analysis revealed different risk factors associated with progression to ESRD such as high serum creatinine at diagnosis, increasing age, need for hemodialysis at diagnosis, positive ANCA, presence of significant proteinuria (>1 g/24h), low serum C3 and high rate of serious infections during follow-up. By multivariate analysis, only serum creatinine at diagnosis (HR = 16.8, 95% CI = 1.354 − 208.62, *p* = 0.028) and low serum C3 (HR = 2.492, 95% CI = 1.537–11.537, *p* = 0.044) remained statistically significant (Table 4).

**Table 4.** Uni- and multivariate Cox regression analysis of variables associated with ESRD progression.

	Univariate analysis		Multivariate analysis	
	HR (95% CI)	*p-*value	HR (95% CI)	*p-*value
Age	1.113 (1.038–1.192)	0.002	0.632 (0.392–1.019)	0.060
**Serum creatinine at diagnosis**	2.739 (1.759–4.264)	0.000	**16.8 (1.354–208.62)**	**0.028**
Hemodialysis at diagnosis	37.5 (7.273–193.937)	0.000	11.49 (0.196–67.25)	0.240
ANCA (+) status	5.901 (1.906–18.267)	0.02	0.731 (0.079–6.756)	0.782
Sclerotic pattern (kidney biopsy)	5.789 (0.957–35.019)	0.056		
Proteinuria >1 g/24h	5.949 (1.262–28.044)	0.024	13.046 (0.759–224.270)	0.077
**Low serum C3 (< 75 mg/dL)**	9.82 (2.592–37.208)	0.001	**2.492 (1.537–11.567)**	**0.044**
Positive IF for C3 (kidney biopsy)	1.16 (0.272–4.948)	0.840		
Serious infections during follow-up	4.653 (1.107–19.558)	0.036	2.618 (0.294–23.310)	0.388

Cox regression analysis, *p* value refers to likelihood ratio test, 95% CI, 95% confidence interval.

ANCA, antineutrophil cytoplasmic antibodies; C3, complement 3 level; IF, immunofluorescence. Statistical significant differences are shown in bold (*p* < 0.05).

## Discussion

This study highlights a potential association between low serum C3 levels at diagnosis and renal severity as well as renal prognosis in patients with AAV. This is one of the few studies showing that serum hypocomplementemia at diagnosis may be an independent prognostic factor for ESRD progression in patients with AAV.

A role for complement activation in the pathogenesis of ANCA disease is supported by *in vitro* studies [6,7], histologic data from kidney biopsies showing complement deposition [8] as well as data from animal models and humans [9,10] indicating that inhibition of complement may be therapeutically efficacious in AAVs. These studies demonstrated that the complement activation by alternative pathway and particularly the receptor C5a (C5aR) is important in the maintenance of the inflammatory process. Two inhibitors of the complement pathway are in clinical development for ANCA vasculitis: avacopan, an oral C5a receptor inhibitor, that has demonstrated efficacy, safety and steroid sparing in two Phase II trials, being it a promising future strategy and eculizumab, a monoclonal antibody that targeted against complement C5, which inhibits the cleavage of C5 into C5a and C5b [9,10]. Furthermore, the positive effect of plasma exchange in severe AAV is hypothetically due, at least in part, to the removal of activated complement factors and of chemotaxins [10]. However, whether or not serum or kidney deposited C3 can be used as markers of disease severity or prognosis (ESRD/death) in patients with AAV is unclear.

Low serum C3 levels have been reported in 4-35% of patients with AAV [3,11–17] (Table 5). In our cohort 23% of patients were hypocomplementemic at diagnosis. There are conflicting literature data regarding the potential association between C3 levels and severity of renal disease. Some studies did not find a significant correlation between hypocomplementemia and creatinine levels [3,13,15–17] whereas others did [11,12,14]. Recently, Garcia et al. [14] in a cohort of 93 patients described low C3 levels in 11% and that was associated significantly not only with higher creatinine levels, but as well with proteinuria > 1g/day and active urinary sediment. Similarly, Villacorta et al. [16] on 111 patients with AAV also described a significant correlation between hypocomplementemia and the need for dialysis at diagnosis.

**Table 5.** Association between low C3 levels and C3 IF in AAV and renal disease and outcome.

References	Patients	Proportion of low C3	IF C3	Impact of low serum C3
Garcia et al. [14]	93	11.1% (7/63)	13.3% (4/30)	Higher frequency of renal involvement (*p* = 0.036)
Fukui et al. [3]	81	20% (16/81)	No data	More often skin lesions (*p* = 0.002), TMA (*p* = 0.07), Diffuse alveolar hemorrhage (*p* = 0.006), mortality (*p* = 0.033)
Villacorta et al. [16]	111	35% (39/111)	25.2% (28/111)	Higher creatinine level at diagnosis (p > 0.05)
				Need for dialysis at onset (*p* = 0.04)
				Lower response to treatment (*p* = 0.007)
				Poorer renal and overall survival (*p* = 0.01)
Deshayes et al. [15]	76	5% (4/76)	No data	Treated more often with PEX (*p* = 0.04)
				Poorer renal (*p* < 0.001) and overall survival (*p* = 0.0011)
Crnogorac et al.[17]	75	12% (9/75)	No data	Lower overall and renal survival (*p* = 0.016)
Manenti et al.[13]	46	35% (16/46)	No data	Older age (*p* = 0.04)
				Lower eGFR at diagnosis (p > 0.05)
				More often ESRD (*p* = 0.02)
Augusto et al.[11]	45	49% (22/45)	No data	Higher creatinine at diagnosis (*p* = 0.008)
				Lower C4 level (*p* = 0.008)
				Poorer renal (*p* = 0.001) and overall survival (*p* = 0.047)
Molad et al.[12]	30	20% (6/30)	20% (1/5)	Older age (*p* = 0.009)
				Lower eGFR at diagnosis (*p* = 0.03)
				Poorer renal (*p* = 0.002) and overall survival (*p* = 0.02)
Haas et al.[25]	126	No data	54% (68/126)	Higher proteinuria (*p* < 0.0001)
				Higher percentage of glomeruli with crescents (*p* = 0.06)
Chen et al.[24]	112	0.8% (4/112)	33% (37/112)	Higher proteinuria (*p* < 0.01)
				Higher creatinine at diagnosis (*p* < 0.05)
				Need for dialysis at onset (*p* < 0.05)
Scaglioni et al.[23]	53	No data	26.4% (14/53)	Higher proteinuria (*p* = 0.0036)

C3, complement 3; IF, immunofluorescence; TMA, thrombotic microangiopathy; PEX, plasma-exchange; ESRD, end-stage renal disease; C4, complement 4, *p* < 0.05 statistical significant.

In our patient cohort, low C3 levels were associated with more severe renal disease (as evidenced by high serum creatinine and need for hemodialysis) and worse renal prognosis (progression to ESRD). Therefore, these findings support the hypothesis of systemic activation of the complement in a proportion of patients with AAV and this could have clinical and prognostic implications.

There has been some evidence from previous studies that serum creatinine at diagnosis could be related to worse prognosis in patients with AAV [18–22]. In our cohort, we found a strong association between serum creatinine at diagnosis and progression to ESRD. The novel finding of our study was that low serum C3 levels were an independent predictor of ESRD progression. Few studies have shown that hypocomplementemia at the presentation of the AAV was associated with worse renal prognosis [11–13,15,17]. Crnogorac et al. [17] included 75 patients with AAV and renal involvement and suggested that low serum C3 at diagnosis could be associated with worse renal prognosis (ESRD). On contrary, Augusto et al. [11] on 45 patients found a significant correlation between even lower normal C3 values at diagnosis with ESRD related mainly to histologic data (crescentic/mixed histologic form).

In the literature, deposits of complement in kidney biopsies have been reported in 25–54% of patients with AAV [16,23,24,25] (Table 5). Hass et al. [25] studied 126 biopsies and found in 54% of them immune complex deposits on electron microscopy. In these studies, C3 deposition was associated with proteinuria [23–25], higher creatinine [24], lower C3 [13] and certain parameters of renal histopathology, such as higher percentage of crescent formation [24,25].

In our study, more than half of patients showed C3 renal deposition. These patients had more often low serum C3 levels, a mixed histologic pattern in kidney biopsies and presented more frequently serious infections during follow-up. However, there was no correlation between kidney C3 deposition and AAV disease severity and prognosis (ESRD).

Our study has certain limitations including its retrospective design, the small number of included patients and the missing data regarding kidney biopsies. On contrary, this is one of the few studies in the literature where complement serum levels and kidney deposition were examined as markers of disease severity and prognosis during long-term follow-up (∼4.5 years).

Our findings are novel indicating that the subgroup of patients with AAV with low C3 at diagnosis has a more severe disease and a higher risk for progression to ESRD. Consequently, more multicentric research studies are needed to confirm our study findings, so this should be taken into account when the appropriate therapeutic and monitoring strategies are designed.

## Ethical policy and institutional review board statement

This research was approved by the Hippokration General Hospital’s institutional review board and consent of all patients for their retrospective chart review was obtained.

## Supplementary Material

Supplemental Material
