# Supplementary material for: Hypocomplementemia is associated with more severe renal disease and worse renal outcomes in patients with ANCA-associated vasculitis: a retrospective cohort study
Source: Ren Fail. 2020 Aug 13;42(1):845–52. doi: 10.1080/0886022X.2020.1803086 (PMC7472496; doi:10.1080/0886022X.2020.1803086)
Supplement: Supplemental Material [file IRNF_A_1803086_SM9783.pdf]

Supp Table 1

C3 and C4 levels value at diagnosis

**Total n=47**	**C3 level mg/dl (normal range 75-180)**	**C4 level mg/dl (normal range 10-40)**
1	119	17
2	118	24.9
3	153	36.2
4	152	35.5
5	131	45.1
**6**	**74**	**19.7**
7	150	50
8	108	27.5
9	160	41.8
10	149	27
11	79	17.6
12	133	27
13	156	27.2
14	128	32.4
15	164	24.9
16	95	21.4
17	159	17.4
18	150	30
19	111	37.2
20	144	31
21	115	46
22	138	23
23	106	16.1
24	131	24

25	100	38
26	109	21.2
27	82	18.2
28	155	32.9
**29**	**48**	**8.2**
**30**	**58**	**18.7**
**31**	**58**	**16.2**
32	112	25.9
33	116	25.4
**34**	**65**	**11.9**
35	168	48.5
36	136	39.8
37	118	32.7
38	131	36.1
39	118	32.7
40	131	36.1
**41**	**67**	**21**
**42**	**58**	**13**
43	110	18
**44**	**72**	**10**
**45**	**56**	**14.8**
**46**	**63**	**20.7**
**47**	**74**	**14.1**

C3, complement 3; C4, complement 4. Patients with hypocomplementemia are marked in bold.
